# Anti-cancer characteristics and ototoxicity of platinum(II) amine complexes with only one leaving ligand

**DOI:** 10.1371/journal.pone.0192505

**Published:** 2018-03-07

**Authors:** Jerry D. Monroe, Heidi L. Hruska, Hannah K. Ruggles, Kevin M. Williams, Michael E. Smith

**Affiliations:** 1 Department of Biology, Western Kentucky University, 1906 College Heights Boulevard, Bowling Green, KY, United States of America; 2 Department of Chemistry, Western Kentucky University, 1906 College Heights Boulevard, Bowling Green, KY, United States of America; Shiraz University, ISLAMIC REPUBLIC OF IRAN

## Abstract

Unlike cisplatin, which forms bifunctional DNA adducts, monofunctional platinum(II) complexes bind only one strand of DNA and might target cancer without causing auditory side-effects associated with cisplatin treatment. We synthesized the monofunctional triamine-ligated platinum(II) complexes, Pt(diethylenetriamine)Cl, [Pt(dien)Cl]^+^, and Pt(N,N-diethyldiethylenetriamine)Cl, [Pt(Et_2_dien)Cl]^+^, and the monofunctional heterocyclic-ligated platinum(II) complexes, pyriplatin and phenanthriplatin, and compared their 5’-GMP binding rates, cellular compartmental distribution and cellular viability effects. A zebrafish inner ear model was used to determine if the monofunctional complexes and cisplatin caused hearing threshold shifts and reduced auditory hair cell density. The four monofunctional complexes had varied relative GMP binding rates, but similar cytosolic and nuclear compartmental uptake in three cancer cell lines (A549, Caco2, HTB16) and a control cell line (IMR90). Phenanthriplatin had the strongest effect against cellular viability, comparable to cisplatin, followed by [Pt(Et_2_dien)Cl]^+^, pyriplatin and [Pt(dien)Cl]^+^. Phenanthriplatin also produced the highest hearing threshold shifts followed by [Pt(dien)Cl]^+^, [Pt(Et_2_dien)Cl]^+^, cisplatin and pyriplatin. Hair cell counts taken from four regions of the zebrafish saccule showed that cisplatin significantly reduced hair cell density in three regions and phenanthriplatin in only one region, with the other complexes having no significant effect. Utricular hair cell density was not reduced by any of the compounds. Our results suggest that placing greater steric hindrance *cis* to one side of the platinum coordinating center in monofunctional complexes promotes efficient targeting of the nuclear compartment and guanosine residues, and may be responsible for reducing cancer cell viability. Also, the monofunctional compounds caused hearing threshold shifts with minimal effect on hair cell density, which suggests that they may affect different pathways than cisplatin.

## Introduction

A major side-effect of platinum-based anticancer drugs is damage to auditory hair cells [1–4]. These specialized cells transduce sound stimuli into a neural signal but can be damaged or destroyed by chemical compounds known as ototoxins. Destroyed hair cells cannot regenerate in mammals leading to permanent hearing loss [1–4]. Patients treated with platinum-based chemotherapy drugs often exhibit hearing and balance deficits, with some studies reporting incidence rates ranging from 13 to 95% from administration of cisplatin [4]. While the cancer treatment benefits obtained from platinum-containing drugs is evident, developing new forms of these drugs that possess anti-cancer activity without causing hearing loss is greatly needed.

Platinum-based drugs currently approved by the FDA, e.g., cisplatin, oxaliplatin and carboplatin, have diamine ligands with either a bidentate chelating ligand or two ligands that can be replaced by water through aquation reactions [5]. These chemotherapeutic agents form bonds with DNA that promote cytotoxicity [6–8]. Once in the cell, these drugs undergo aquation of their leaving groups enabling them to react with DNA and proteins [6, 9–10]. They usually react with guanine bases and typically form bifunctional 1,2-intrastrand cross-links [11]. This crosslink causes DNA to distort, forming binding sites for proteins, e.g., HMG1 and HMG2, which activate apoptotic mechanisms [11]. An unfortunate consequence is often hearing loss caused by apoptotic damage to auditory hair cells [12–16].

**Fig 1:**
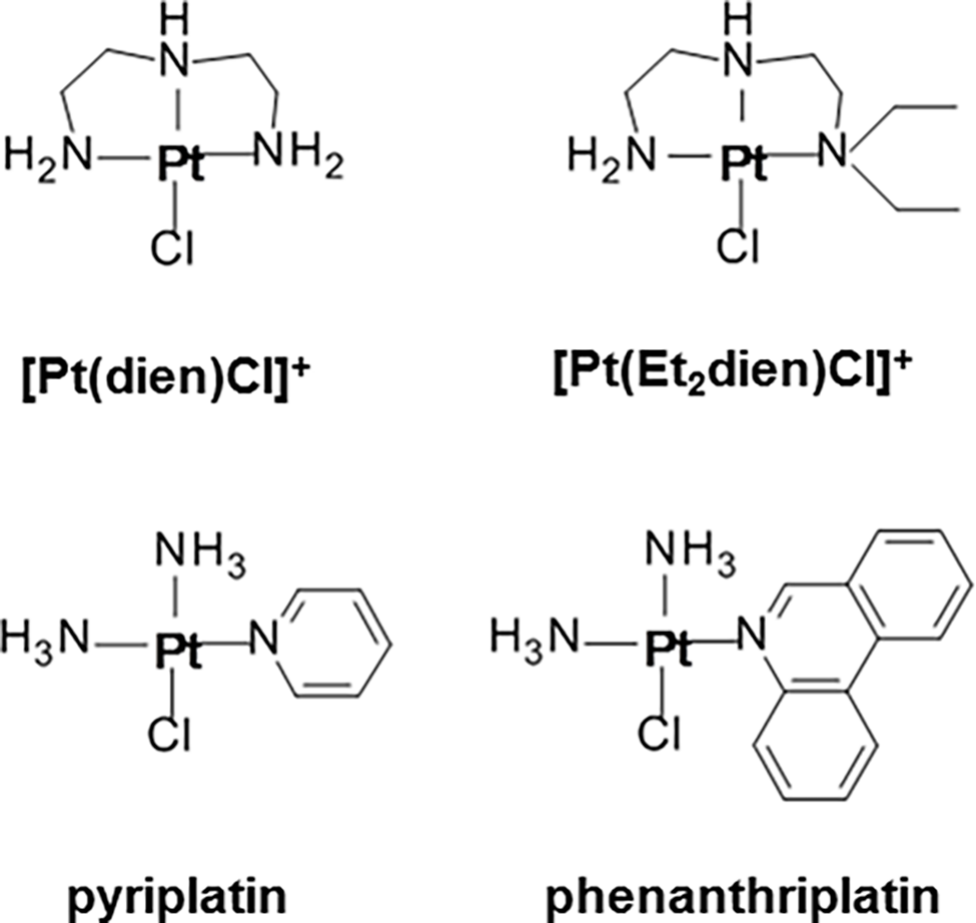
Monofunctinal platinum(II) complexes investigated in this project. [Pt(dien)Cl]^+^ and [Pt(Et_2_dien)Cl]^+^ are triamine ligated compounds with similar steric properties to pyriplatin and phenanthriplatin. Pyriplatin and phenanthriplatin are heterocyclic compounds that reduce cancer cell viability. Abbreviations: dien = diethylenetriamine, Et_2_dien = N,N diethyldiethylene-triamine.

Among several platinum-based drug candidates currently in clinical trials, new structural design strategies have emerged. One strategy involves increasing steric bulk to prevent drug inactivation by binding to intracellular thiols [5]. Another strategy is to develop drugs that form monofunctional adducts with DNA without distorting it as is done by traditional platinum complexes [5]. A number of recently synthesized platinum complexes have increased steric hindrance and form monofunctional adducts [5, 17–20]. Two platinum(II) complexes with heterocyclic ligands, pyriplatin and phenanthriplatin (Fig 1), form monofunctional adducts with DNA and significantly reduce cancer cell viability [19; 21–22]. The two triamine-ligated compounds, [Pt(dien)Cl]^+^ and [Pt(Et_2_dien)Cl]^+^ (Fig 1), which are variants of diethylenetriamine, are similar to pyriplatin and phenanthriplatin in that they have increased steric hindrance around the platinum coordinating center and form monofunctional adducts [23–25]. Some new heterocyclic-ligated platinum(II) compounds, unlike diamine-ligated drugs, leave DNA repair mechanisms intact [20]. Interference with these mechanisms is an initial step in the activation of apoptotic pathways associated with ototoxicity [11]. Therefore, these heterocyclic- and triamine-ligated platinum(II) compounds have structural and functional properties that could cause them to signal through different apoptotic pathways and produce reduced side-effects.

Here, we comparatively evaluated the 5’-GMP binding rates, cancer cell compartmental uptake, and effect on cancer and control cell viability of pyriplatin, phenanthriplatin, [Pt(dien)Cl]^+^ and [Pt(Et_2_dien)Cl]^+^. Then, we determined if these complexes produced hearing threshold shifts and auditory epithelial hair cell loss in a zebrafish inner ear model using the auditory evoked potential (AEP) and fluorescent cell staining techniques. Our results suggest that triamine-ligated platinum complexes with increased steric bulk *cis* to the coordinating center exhibit properties competitive with those of heterocyclic-ligated platinum complexes. We also found that cisplatin, and the monofunctional platinum(II) complexes, with the exception of pyriplatin, cause zebrafish hearing threshold shifts at most test frequencies 48 hours following treatment. However, the monofunctional compounds, except phenanthriplatin in one region, did not decrease hair cell density in the saccule and utricle sensory epithelia 48 hours following treatment; whereas, cisplatin causes a significant reduction in most regions of the saccule.

## Material and methods

### Synthesis of platinum complexes

Phenanthriplatin and pyriplatin were synthesized similar to the method described in [19]. Cisplatin (300 mg) was dissolved in 15 mL of DMF and AgNO_3_ (168 mg) was added; after 24 hours of stirring, the sample was filtered to remove AgCl. The amine (phenanthridine and pyridine, respectively) were added (0.9 mole equivalent), and the reaction was stirred for 24 hours at 55°C. The solvent was removed and 30 mL of methanol was added. After filtering to remove the cisplatin, 100 mL of diethylether was added while stirring vigorously to precipitate the compound as a solid. The solid was collected and washed with diethylether. To further purify the compound, the sample was redissolved in methanol and precipitated by dropwise addition into vigorously stirred ether. The sample was collected and dried, and the purity was checked by NMR on a 500 MHz instrument (JEOL, Tokyo, Japan) and compared with literature values. ^1^H NMR samples in D_2_O referenced to the HOD residual signal were corrected for temperature [26]. All compounds used for synthesis of the platinum compounds were from Sigma-Aldrich (St Louis, MO) except for pyridine and dien (Acros, Geel, Belgium).

[Pt(dien)Cl]^+^ and [Pt(Et_2_dien)Cl]^+^ were prepared similarly to the method of [23] for [Pt(dien)Cl]^+^. Potassium tetrachloroplatinate (1 g) was dissolved in 30 mL of H_2_O, and 1 mL of the amine was added. The pH was lowered to ≈3 with HCl, and the solution was refluxed for at least 6 h. The volume was reduced to ≈4 mL, and the yellow precipitate was collected and washed with ethanol. As necessary, the compound was purified by recrystallization by dissolving in a small amount of hot water and cooling. [Pt(Et_2_dien)Cl]^+ 1^H NMR (500 MHz, D_2_O): 1.29 ppm (3H, t), 1.32 ppm (3H, t), 2.59 ppm, (3H, overlapping m), 2.77 ppm (1H, m), 3.12 ppm (1H, m), 3.27 ppm (4H, overlapping m), 3.37 (1H, m), 3.46 ppm (1H, m), 3.89 ppm (1H, m).

### Kinetic 5'-GMP assays

The reaction of each platinum compound with 5'-GMP (Acros) was monitored using ^1^H NMR spectroscopy to observe the disappearance of the H8 signal of the unreacted 5'-GMP at ≈8 ppm and the appearance of a product signal ≈0.5–1.0 ppm downfield of the unreacted 5'-GMP. The reaction was conducted with excess platinum compound present in each case. A 10 mM solution of 5'-GMP and a 10–20 mM solution of the platinum(II) compound were prepared separately in D_2_O. The pH (uncorrected for the effects of deuterium) of each solution was adjusted to 7, and the samples were equilibrated to 25°C in a water bath. Aliquots of the two solutions were combined and placed in an NMR tube; typically, the final concentrations of platinum was 5–18 mM and the concentration of 5'-GMP was 1 mM (except for the [Pt(dien)Cl]^+^ reaction, where it was 0.5 mM). Samples were maintained at 25°C throughout the NMR experiment. The H8 resonances were integrated and the data was fitted to an equation that was first order in each reactant using DynaFit to determine rate constants. At least two runs were conducted for each platinum compound using fresh samples.

### Cell culture

Cell lines were obtained from American Type Culture Collection (ATCC, Manassas, VA). The A549 (ATCC: CCL-185) lung cancer cell line was grown in F12K media with 10% FBS and 1% penicillin/streptomycin supplementation. Both the U-138 MG (ATCC: HTB-16) glioblastoma cancer cell line and IMR-90 (ATCC: CCL-186) lung fibroblast control cell line were grown in Eagle’s Minimum Essential Medium (EMEM) with supplementation as in A549. Caco-2 (ATCC: HTB-37) colorectal cancer was grown in EMEM with 20% FBS and 1% penicillin/streptomycin. All cell lines were incubated at 37°C in 5% CO_2_ with passaging every 4–7 days. Cell culture media and supplements were obtained from ATCC, Atlanta Biologicals (Flowery Branch, GA), Mediatech (Tewksbury, MA), Fisher Scientific (Waltham, MA), Gibco (Gaithersburg, MD) and Sigma-Aldrich.

### Determination of cellular platinum distribution

Intracellular platinum uptake and distribution was performed using a methodology adapted from [19]. Approximately 10^6^ cells were seeded in a 10 cm diameter Petri dish in triplicate in culture medium and incubated for 24 hrs. Cells were then treated with 5 μM of platinum compound at 37°C in 5% CO_2_ for 3 hrs. Then, medium was removed and cells were washed with 2 mL of ice-cold PBS 3X to remove excess platinum. Cells were then detached using 1 mL trypsin, and transferred to a centrifuge tube with addition of 0.5 mL PBS. Next, centrifugation was performed at 200 x g and 4°C for 20 min. Cell pellets were resuspended in 200 μL of ice-cold lysis buffer (1.0 mM dithiothreitol (DTT), 1.0 mM phenylmethanesulfonylfluoride (PMSF), 10 mM KCl, and 10 mM MgCl_2_, pH 7.5) and then cooled for 15 min in an ice bath. Cells were then centrifuged at 450 x g and 4°C for 20 min. Supernatant was then removed and pellets resuspended in 150 μL of ice-cold lysis buffer. Cell membranes were then lysed by passing them 10X through a syringe with a 28-gauge needle. The resulting suspension was centrifuged at 11,000 x g for 20 min at 4°C, and the supernatant was retained as the cytosolic fraction. The remaining pellet, the nuclear material, was resuspended in 150 μL of extraction buffer (1.0 mM DTT, 1.0 mM PMSF, 1.5 mM MgCl_2_, 0.2 M ethylenediaminetetraacetic acid (EDTA), 0.42 M NaCl, and 25% glycerol, pH 7.9) and was then lysed by passing it 10X through a 28-gauge syringe. The lysate was then shaken at 1,000 rpm for 1 h at 4°C and centrifuged at 20,000 x g for 10 min at 4°C. The supernatant was then collected as the nuclear fraction. Platinum concentrations in all fractions were determined by atomic absorption spectroscopy using a Varian (Palo Alto, CA) AA240 FS Graphite Furnace AA; a calibration curve was established using a platinum standard solution. A wavelength of 265.9 nm was utilized for all measurements. Reagents were obtained from ATCC, Atlanta Biologicals, Mediatech, Fisher Scientific, Gibco and Sigma-Aldrich.

### Effect of platinum complexes on cellular viability

The MTT (3-(4,5-dimethylthiazol-2-yl)-2,5-diphenyltetrazolium bromide) cellular viability assay was performed in all three cancer cell lines and the non-cancer cell line. Following a protocol adapted from [19], cells were seeded at 5,000 cells per well in 96-well plates and incubated at 37°C in 5% CO_2_ in F-12K Medium or EMEM with FBS supplementation and 1% penicillin/streptomycin for 24 hours. Then, wells were treated in replicates of six according to a concentration series (500, 50, 5, 0.5, 0.05 μM) of specific platinum complexes for 72 hours. A negative control (media only), positive control (Triton X-100) and series of blanks were simultaneously performed. The MTT assay was run for 2 hours, then well contents were rapidly evacuated out and all wells were treated with MTT solubilization solution [10% Triton X-100 in acidic (0.1N HCl) isopropanol] for 15 minutes before the plates were read using a BioTek Synergy HT microplate plate reader (570 nm and 690 nm absorbance wavelengths). Reagents, cell culture media and supplements were obtained from ATCC, Atlanta Biologicals, Mediatech, Fisher Scientific, Gibco and Sigma-Aldrich.

### Zebrafish maintenance

Zebrafish (*Danio rerio*) were purchased from a commercial supplier (Segrest Farms, Gibsonton, FL) and maintained in the animal facility of Western Kentucky University according to a protocol approved by the Institutional Animal Care and Use Committee. All zebrafish used in this study were a mix of male and female adult animals at least 6 months of age and were maintained according to standard methods [27].

### Microinjection

Zebrafish were sedated with tricaine methanesulfonate (MS-222, Argent, Redmond, WA), weighed, and then intraperitoneally microinjected with 25 mg/kg body weight of platinum test compound or an equivalent volume of vehicle (0.9% NaCl) control using a 35 gauge syringe operated by a Micro4 microsyringe pump controller (World Precision Instruments, Sarasota, FL). After injection, fish were returned to holding tanks for either 24 or 48 hours and then subjected to hearing tests followed by hair cell quantification analysis.

### Auditory evoked potential testing

Auditory evoked potential (AEP) testing was performed on individual fish according to standard protocols [28–33]. Fish were anaesthetized with MS-222, restrained in a mesh harness, and suspended under water in a 19 L vessel such that the head was approximately 6 cm below the surface and 22 cm above an underwater speaker. After harness placement was complete, three 27 gauge stainless steel electrodes (Rochester Electro-Medical, Inc., Lutz, FL) were inserted approximately 2 mm subdermally to record AEPs. A reference electrode was inserted into the medial dorsal surface of the head, a recording electrode directly over the brainstem and a ground electrode in the tail musculature. Sound stimuli (tone pips) were presented to each fish at 8 frequencies (100, 250, 400, 600, 800, 1000, 1500 and 3000 Hz) and AEP waveforms were collected using a TDT System III physiology apparatus (Tucker-Davis Technologies Inc., Alachua, FL) running SigGen and BioSig software. Auditory stimuli were passed through a P1000 power amplifier (Hafler, Port Coquitlam, BC) and presented with a University Sound UW-30 underwater speaker (Electro-Voice, Fairport, NY). Sound pressure levels were confirmed using a Type 10CT hydrophone (G.R.A.S., Holte, Denmark) placed adjacent to the mesh harness. Auditory thresholds were determined in 5 dB increments by visual inspection of AEP waveforms.

### Hair cell quantification

Hair cell counts were performed from specific regions of the zebrafish saccule and utricle using established procedures [32–35]. First, zebrafish were euthanized using an overdose of MS-222 per American Veterinary Medical Association (AVMA) recommendation, the lower jaw was removed and the head was fixed in 4% paraformaldehyde (Alfa-Aesar, Ward Hill, MA) overnight at 4°C. Prior to dissection, heads were rinsed three times for 10 minutes each at room temperature in phosphate buffer (pH 7.4, LabChem, Pittsburgh, PA) and the inner ear epithelia (saccule and utricle) were then carefully dissected out and trimmed. Dissected endorgans were first stained with Alexa Fluor 488-conjugated phalloidin (1:100; Life Technologies, Eugene, OR) for 30 minutes to label filamentous actin (F-actin) in stereociliary bundles. Tissue samples were then placed on glass slides and nuclei were labelled with Prolong Gold antifade reagent (4’,6-diamidino-2-phenylindole [DAPI], Life Technologies, Carlsbad, CA) and a cover-slip was placed over the samples for microscopy.

Saccule and utricle samples were viewed through the FITC filter of a Zeiss Axioplan2 epifluorescence microscope (Carl Zeiss, Jena, Germany) at 20X magnification and were then photographed using an AxioCam MRm camera. Cell counts were made from four regions of the saccule (5%, 25%, 50%, 75%) along the rostral-caudal length of the organ by placing a 50 μm x 50 μm box at each location and using the microscope cell counter feature. In the utricular samples, cell counts were made from three equidistant 50 μm x 50 μm boxes in both the extrastriolar and striolar regions.

### IC_50_ and statistical analysis

GraphPad Prism v6 (La Jolla, CA) was used for all statistical analysis. Nuclear fraction platinum uptake as a total percent of combined nuclear and cytosolic uptake was analyzed using a one-way ANOVA (Brown-Forsyth test; p ≤ 0.05). IC_50_ values were calculated in Prism using the sigmoidal, 4PL, x is log(concentration) analysis feature or were calculated from Prism derived values using the equation y = mx + b. MTT assay standard deviation values were calculated using ED50plus v1.0 online software. AEP threshold data were analyzed by two-way ANOVA (Dunnett’s multiple comparisons test; p ≤ 0.05) and hair cell quantification data by two-way ANOVA (Tukey’s multiple comparisons test; p ≤ 0.05). AEP temporary threshold shifts (TTS) were calculated by subtracting the mean vehicle threshold for each frequency from each treatment threshold. TTS data were averaged across frequencies for each treatment and were analyzed by one-way ANOVA (Tukey’s multiple comparisons test; p ≤ 0.05).

## Results

### Kinetic assays

**Fig 2:**
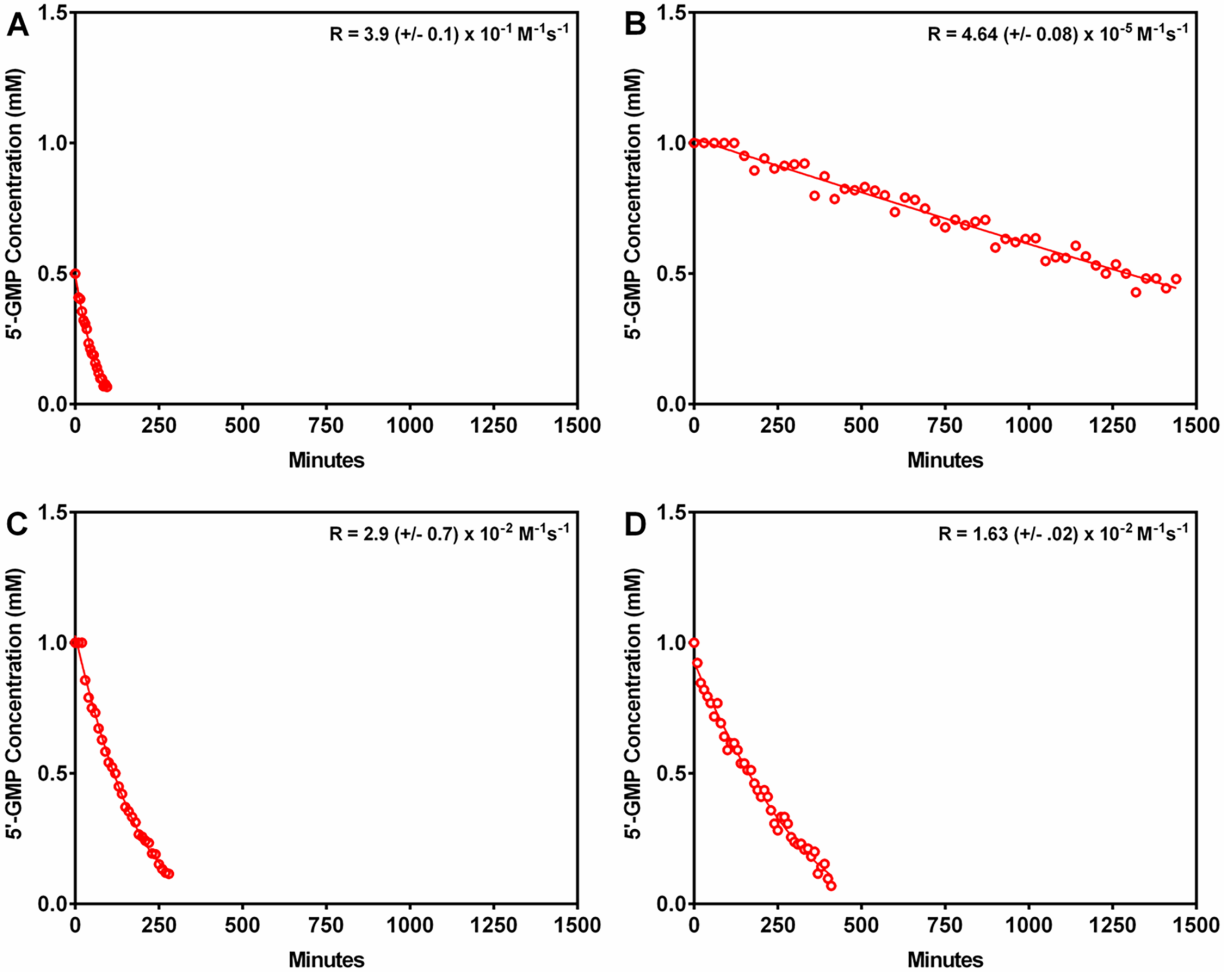
Reaction of 5'-GMP with platinum(II) complexes as measured via ^1^H NMR spectroscopy. A. [Pt(dien)Cl]^+^ (starting Pt concentration 1 mM). B. [Pt(Et_2_dien)Cl]^+^ (starting Pt concentration 18 mM). C. Pyriplatin (starting Pt concentration 5 mM). D. Phenanthriplatin (starting Pt concentration 5 mM). All plots show the reactions of excess platinum(II) complex versus time (minutes). “R” = Rate.

[Pt(dien)Cl]^+^, which would be expected to have no significant steric clashes with an incoming 5'-GMP, was the fastest to react at a rate of 3.9 ± 0.1 x 10^−1^ M^-1^s^-1^ (Table 1, Fig 2). Both pyriplatin (2.9 ± 0.7 x 10^−2^ M^-1^s^-1^) and phenanthriplatin (1.63 ± 0.02 x 10^-2^M^-1^s^-1^) slowed the reaction by a little more than an order of magnitude, with phenanthriplatin showing a ~25 fold reduction in the rate relative to [Pt(dien)Cl]^+^. [Pt(Et_2_dien)Cl]^+^, which has significantly greater steric hindrance relative to the chloride leaving ligand than [Pt(dien)Cl]^+^, phenanthriplatin and pyriplatin, slowed the reaction by nearly 4 orders of magnitude (4.64 ± 0.08 x 10^−5^ M^-1^s^-1^).

**Table 1:** Rate constants for the reaction of 5'-GMP with each platinum(II) complex. The relative rate is a compound’s rate constant divided by the rate constant for [Pt(dien)Cl]^+^, which was the fastest of the four reactions.

Compound	Rate (M^-1^s^-1^)	Relative rate
[Pt(dien)Cl]^+^	3.9 (+/- 0.1) x 10^−1^	1
[Pt(Et_2_dien)Cl]^+^	4.64 (+/- 0.08) x 10^−5^	0.00012
Pyriplatin	2.9 (+/- 0.7) x 10^−2^	0.073
Phenanthriplatin	1.63 (+/- .02) x 10^−2^	0.041

### Compartmental distribution of platinum

**Fig 3:**
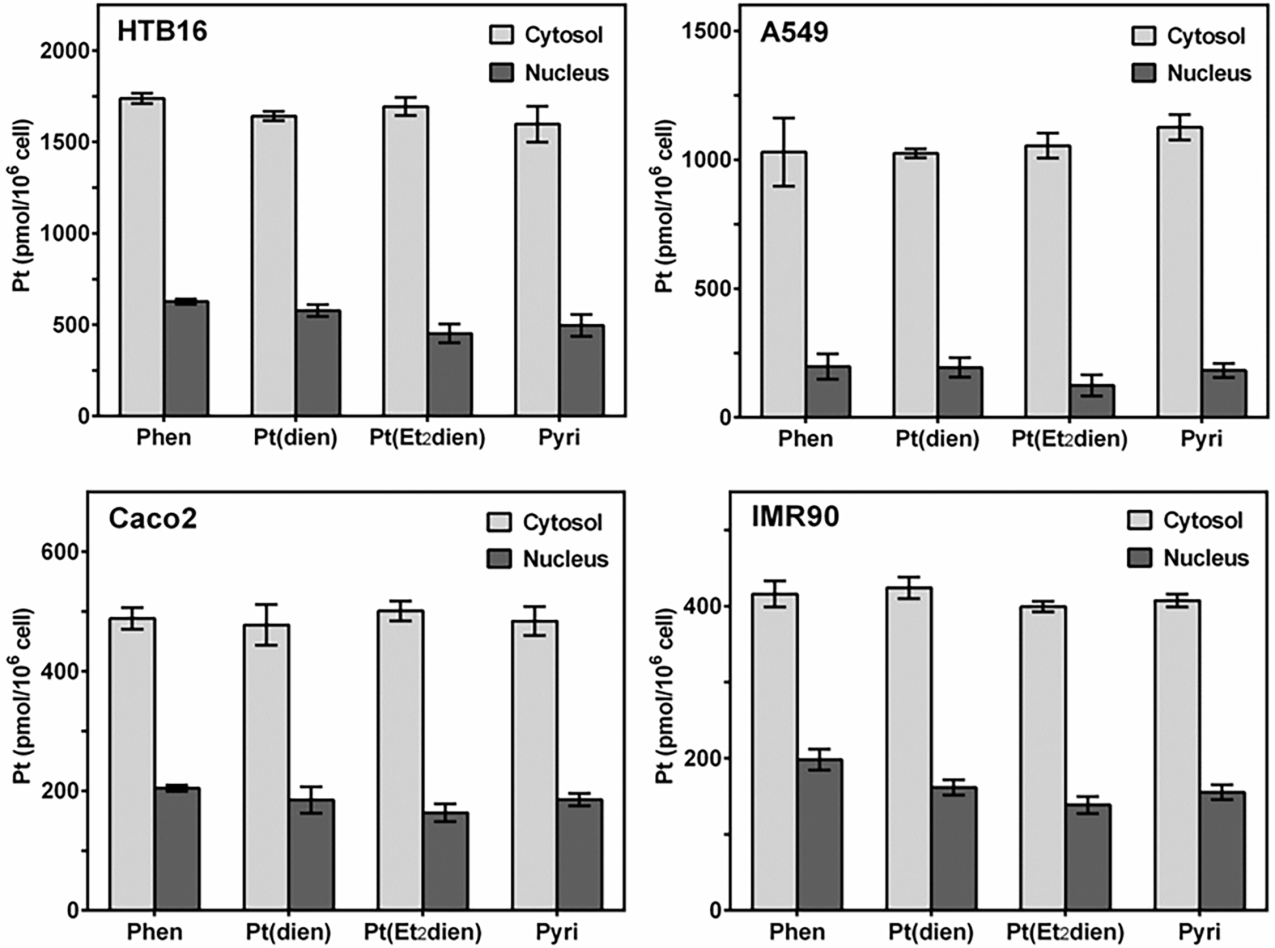
Mean (±SE) compartmental distribution of platinum(II) complexes in cancer and control cell lines. Three cancer cell lines (A549, Caco2 and HTB16), and a non-cancerous fibroblast cell line (IMR90), were treated for 3 hrs with 5 μM of either pyriplatin {Pyri}, phenanthriplatin {Phen}, [Pt(dien)Cl]^+^ {Pt(dien)} or [Pt(Et_2_dien)Cl]^+^ {Pt(Et_2_dien)}. Cellular homogenates were then prepared from each sample (N = 3) and their nuclear and cytosolic fractions were extracted and then quantified for platinum uptake using atomic absorption spectroscopy.

**Fig 4:**
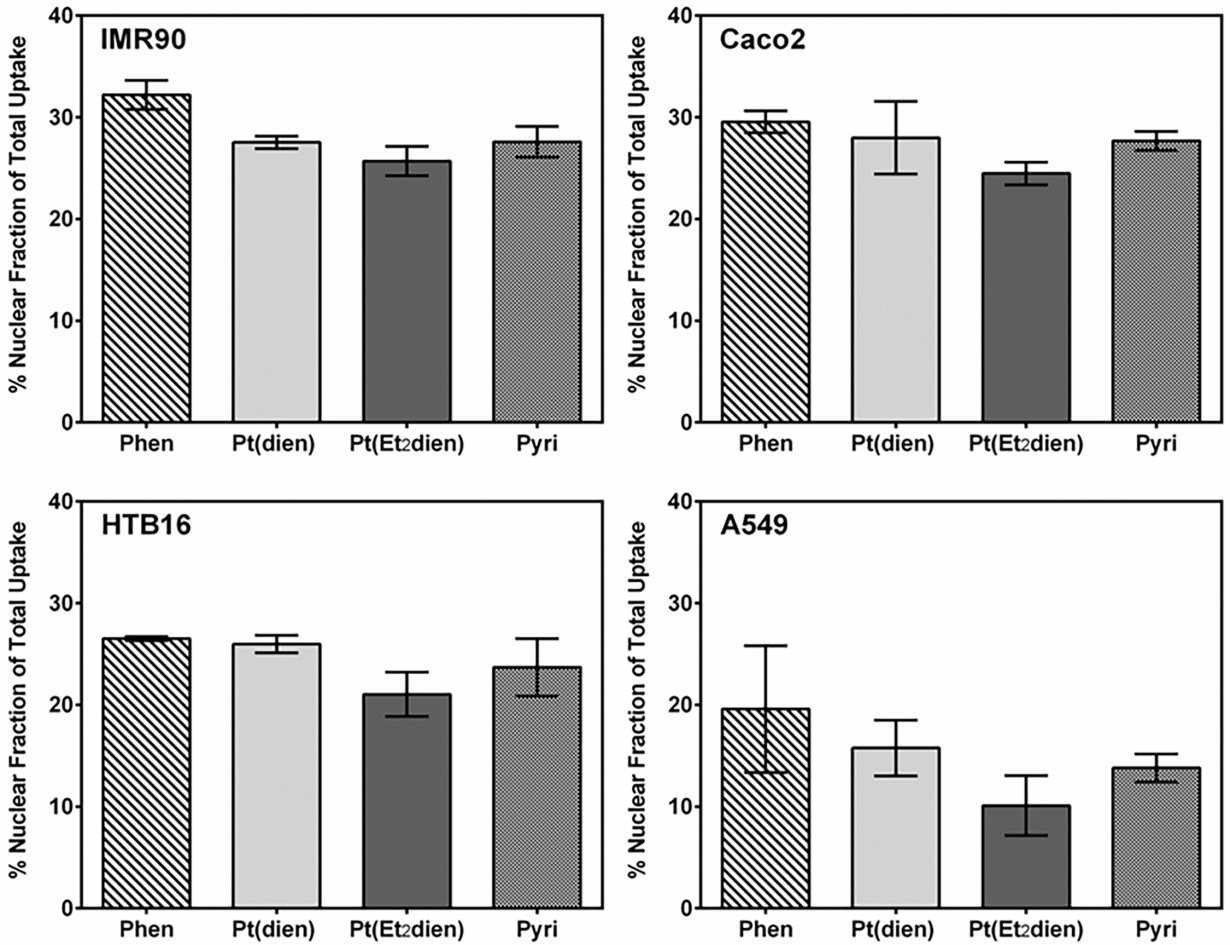
Mean (±SE) nuclear uptake of platinum(II) complexes as a percent of total platinum uptake. Cancer cell and control cultures were treated as described in Fig 3. The nuclear fraction percentage was determined by dividing the nuclear amount of platinum (pmol per 10^6^ cells) by the total amount of platinum (nuclear + cytosolic pmol per 10^6^ cells). N = 3.

Atomic absorption spectroscopy was used to determine platinum uptake in both the cytosolic and nuclear compartments of three cancer cell lines, A549, Caco2 and HTB16, and one non-cancer control line, IMR90. The glioma cell line, HTB16, exhibited higher uptake in both the cytosolic (range: 1597.7 to 1738.2 pmol/10^6^ cells) and nuclear (range: 452.3 to 627.4 pmol/10^6^ cells) compartments for all tested compounds (Fig 3). The non-small cell lung cancer cell line, A549, had an average cytosolic uptake ranging from 1024.7 to 1125.6 pmol/10^6^ cells and nuclear uptake ranging from 123.9 to 197.2 pmol/10^6^ cells. Caco2, a colorectal cancer cell line, exhibited cytosolic uptake ranging from 477.6 to 501.1 pmol/10^6^ cells, roughly half that seen in A549, but had nuclear uptake more similar to A549, from 163.3 to 204.5 pmol/10^6^ cells. The fibroblast control cell line, IMR90, had the lowest amount of cytosolic uptake, ranging from 399.1 to 424.1 pmol/10^6^ cells, but its nuclear uptake ranged from 138.5 to 198.0 pmol/10^6^ cells, similar to both the A549 and Caco2 cell lines. We also investigated whether any of the compounds had greater nuclear uptake as a percentage of total (nuclear + cytosolic) platinum uptake (Fig 4). The percentage of nuclear uptake relative to total uptake ranged from 21.0 to 26.5% in HTB16, 10.1 to 19.6% in A549, 24.5 to 29.6% in Caco2 and 25.7 to 32.2% in IMR90.

### Effect on cancer cell viability

The MTT assay was used to determine the effect of the platinum(II) complexes on cancer and control cell viability. Phenanthriplatin had the strongest anti-cancer activity and produced IC_50_ values comparable to cisplatin (Table 2). Interestingly, both cisplatin and phenanthriplatin strongly reduced the cellular viability of the control cell line, IMR90, and its IC_50_ value was very similar to cancer cell line IC_50_ values. [Pt(dien)Cl]^+^ had the least effect on cellular viability, except against Caco2, where it reduced viability more than [Pt(Et_2_dien)Cl]^+^. [Pt(Et_2_dien)Cl]^+^ reduced control and cancer cell viability more than [Pt(dien)Cl]^+^, with the exception of Caco2, against which it was less effective than any of the other compounds. Pyriplatin reduced control and A549 and HTB16 cancer cell viability to a level intermediate between [Pt(dien)Cl]^+^ and [Pt(Et_2_dien)Cl]^+^; although, as with [Pt(dien)Cl]^+^, pyriplatin effected Caco2 more than [Pt(Et_2_dien)Cl]^+^. Our IC_50_ results for cisplatin, phenanthriplatin and pyriplatin in the A549 cell line are similar to those found for this cell line in [19]. Therefore, our results suggest that the general relationship against cancer and control cell viability of these complexes is: cisplatin ≈ phenanthriplatin < [Pt(Et_2_dien)Cl]^+^ < pyriplatin < [Pt(dien)Cl]^+^.

**Table 2:** IC_50_ values for cisplatin, phenanthriplatin, pyriplatin, [Pt(dien)Cl]^+^ and [Pt(Et_2_dien)Cl]^+^ in cell cultures. Standard deviation values are provided after the ± symbol for each inhibitory concentration value. “NC” indicates non-cancer cell line.

		IC_50_ (μM)				
Cell line	Cancer type	Cisplatin	Pt(dien)Cl^+^	Pt(Et_2_dien)Cl^+^	Phenanthriplatin	Pyriplatin
A549	Lung	9.79 ± 0.63	198.4 ± 4.80	67.30 ± 0.36	0.058 ± 2.07	89.28 ± 0.46
Caco2	Colorectal	0.87 ± 1.77	41.45 ± 0.72	72.48 ± 3.37	1.6 ± 1.22	20.88 ± 0.69
HTB16	Glioma	3.11 ± 0.06	209.1 ± 42.29	48.02 ± 11.80	2.41 ± 0.11	66.29 ± 0.74
IMR90	Fibroblast (NC)	0.53 ± 0.94	56.04 ± 6.10	7.13 ± 0.98	1.24 ± 0.48	16.13 ± 1.13

### Auditory evoked potential testing

**Fig 5:**
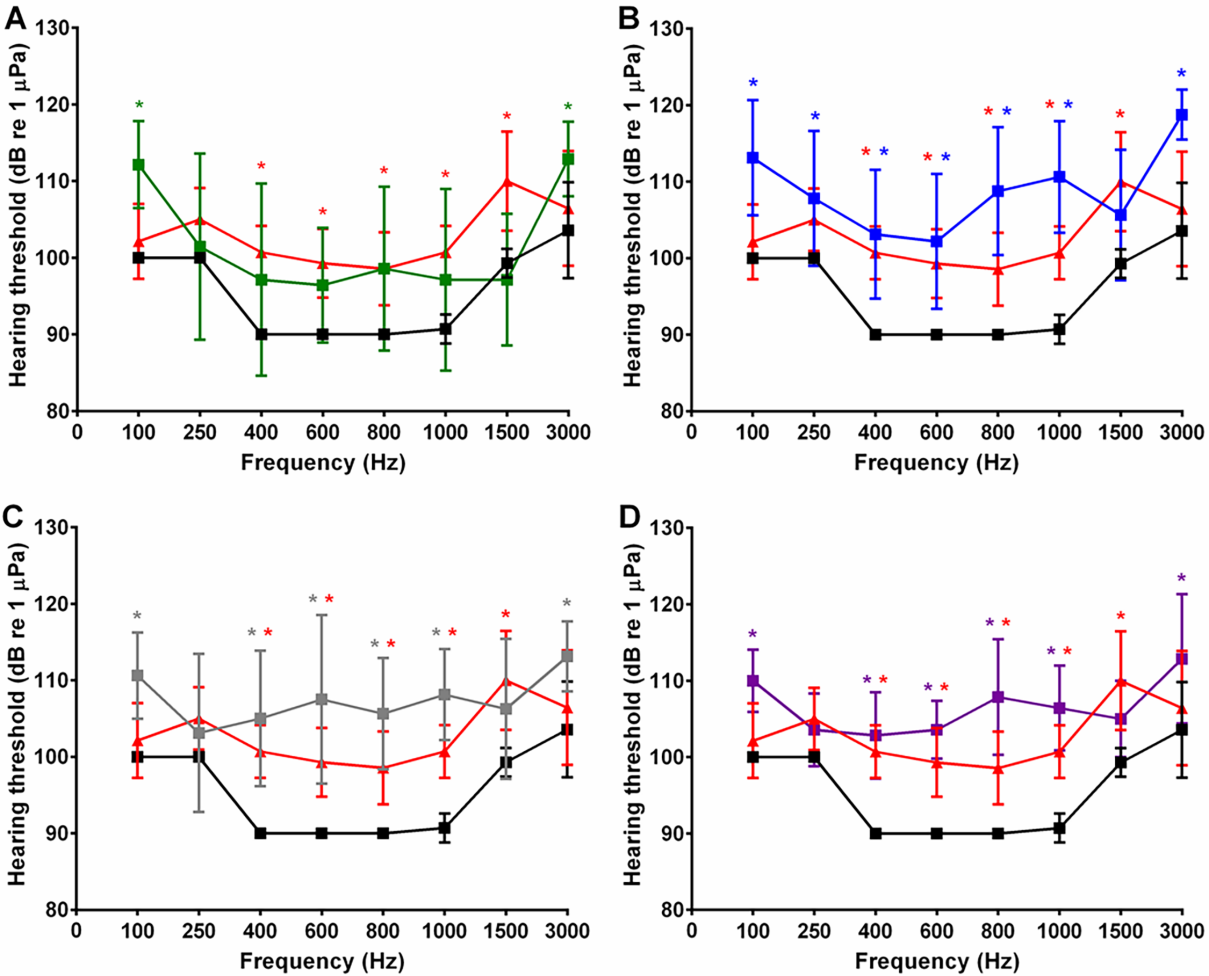
Mean (± SE) AEP hearing thresholds of zebrafish 48 h following injection with either a monofunctional platinum(II) complex, cisplatin, or a vehicle. A.-D. Cisplatin (red) treatment produces threshold shifts between 400 and 1500 Hz; vehicle (black) treatment. A. Pyriplatin (green) treatment only produces a significant threshold shift at 100 Hz. B. Phenanthriplatin (blue), (C) [Pt(dien)Cl]^+^ (grey), and (D) [Pt(Et_2_dien)Cl]^+^ (purple) treatment produces threshold shifts at all but two frequencies (250 and 1500 Hz). N = 8–15; * p ≤ 0.05.

We used the auditory evoked potential (AEP) technique with a zebrafish inner ear model to determine if the platinum(II) complexes caused hearing threshold shifts. When fish were injected for a 24 hour interval with cisplatin and then compared to vehicle-treated fish at this same time interval, threshold shifts were generally absent. However, fish injected with cisplatin for 48 hours had threshold shifts compared to vehicle control-injected fish. We then compared the 48 hour treated cisplatin and vehicle fish with fish injected with phenanthriplatin, pyriplatin, [Pt(dien)Cl]^+^, and [Pt(Et_2_dien)Cl]^+^ for 48 hours. At 48 hours, significant threshold shifts appeared relative to vehicle controls in fish treated with phenanthriplatin, pyriplatin, [Pt(dien)Cl]^+^, and [Pt(Et_2_dien)Cl]^+^ (p < 0.0001; Fig 5). Specifically, *post hoc* tests showed that pyriplatin treatment increased hearing thresholds compared to cisplatin at 1500 Hz and compared to vehicle at 100 Hz (Table 3). Phenanthriplatin treatment produced greater thresholds than cisplatin treatment at four frequencies (100, 800, 1000, 3000 Hz) and caused threshold shifts above those for vehicle treatment at six frequencies (100, 400, 600, 800, 1000, 3000 Hz). Both [Pt(dien)Cl]^+^- and [Pt(Et_2_dien)Cl]^+^-injected fish exhibited higher thresholds relative to controls at the same frequencies as phenanthriplatin-treated fish. [Pt(Et_2_dien)Cl]^+^-injected fish had higher thresholds than cisplatin only at 800 Hz (Table 3).

**Table 3:** Statistical results of Tukey's multiple pairwise comparison tests for zebrafish (*Danio rerio*) AEP threshold data.

	Cisplatin	Pyriplatin		Phenanthriplatin		Pt(dien)Cl^+^		Pt(Et_2_dien)Cl^+^	
Freq(Hz)	Vehicle	Vehicle	Cisplatin	Vehicle	Cisplatin	Vehicle	Cisplatin	Vehicle	Cisplatin
100	ns	*	ns	***	*	*	ns	***	ns
250	ns	ns	ns	ns	ns	ns	ns	ns	ns
400	****	ns	ns	***	ns	****	ns	****	ns
600	***	ns	ns	**	ns	****	ns	****	ns
800	***	ns	ns	****	*	****	ns	****	*
1000	****	ns	ns	****	*	****	ns	****	ns
1500	****	ns	*	ns	ns	ns	ns	ns	ns
3000	ns	ns	ns	****	**	*	ns	**	ns

Hearing thresholds for each platinum compound was tested against thresholds for vehicle controls and cisplatin-injected fish (ns, p > 0.05; * p ≤ 0.05; ** p ≤ 0.01; *** p ≤ 0.001; **** p ≤ 0.0001).

**Fig 6:**
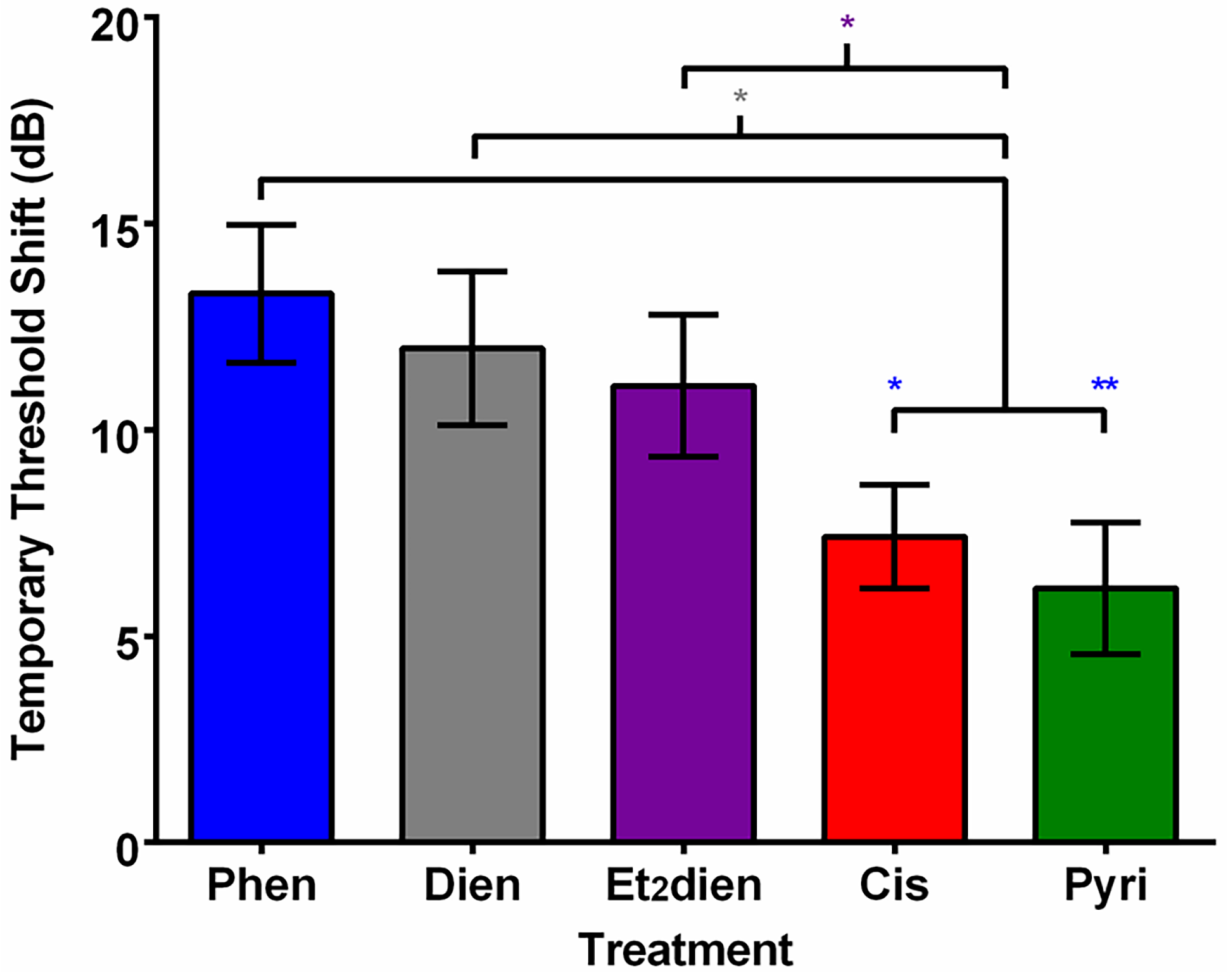
Mean (±SE) temporary threshold shifts of zebrafish 48 hours after injection with either a monofunctional platinum(II) complex or cisplatin. “Phen” = phenanthriplatin; “Dien” = [Pt(dien)Cl]^+^; “Et_2_dien” = [Pt(Et_2_dien)Cl]^+^; “Cis” = cisplatin; “Pyri” = pyriplatin. * p ≤ 0.05; ** p ≤ 0.01. N = 7–8. Color of the asterisks represent the color of the complex being compared to cisplatin and pyriplatin.

After subtracting mean vehicle control thresholds from platinum compound thresholds to calculate temporary threshold shifts (TTS), the overall two-way ANOVA showed significant effects of compound injection treatment (F_4,256_ = 9.77, p < 0.0001) and frequency (F_7,256_ = 8.49, p < 0.0001) on zebrafish TTS, but no significant interaction between the two factors. TTS was greater for fishes injected with phenanthriplatin, [Pt(dien)Cl]^+^, and [Pt(Et_2_dien)Cl]^+^ relative to fishes injected with cisplatin and pyriplatin (Fig 6). Overall, our results suggest that the platinum complexes induce auditory threshold shifts in this series: pyriplatin < cisplatin < [Pt(Et_2_dien)Cl]^+^ ≈ [Pt(dien)Cl]^+^ < phenanthriplatin, with the mean TTS for pyriplatin and phenanthriplatin being, respectively, 6 and 13 dB (Fig 6).

### Hair cell quantification

**Fig 7:**
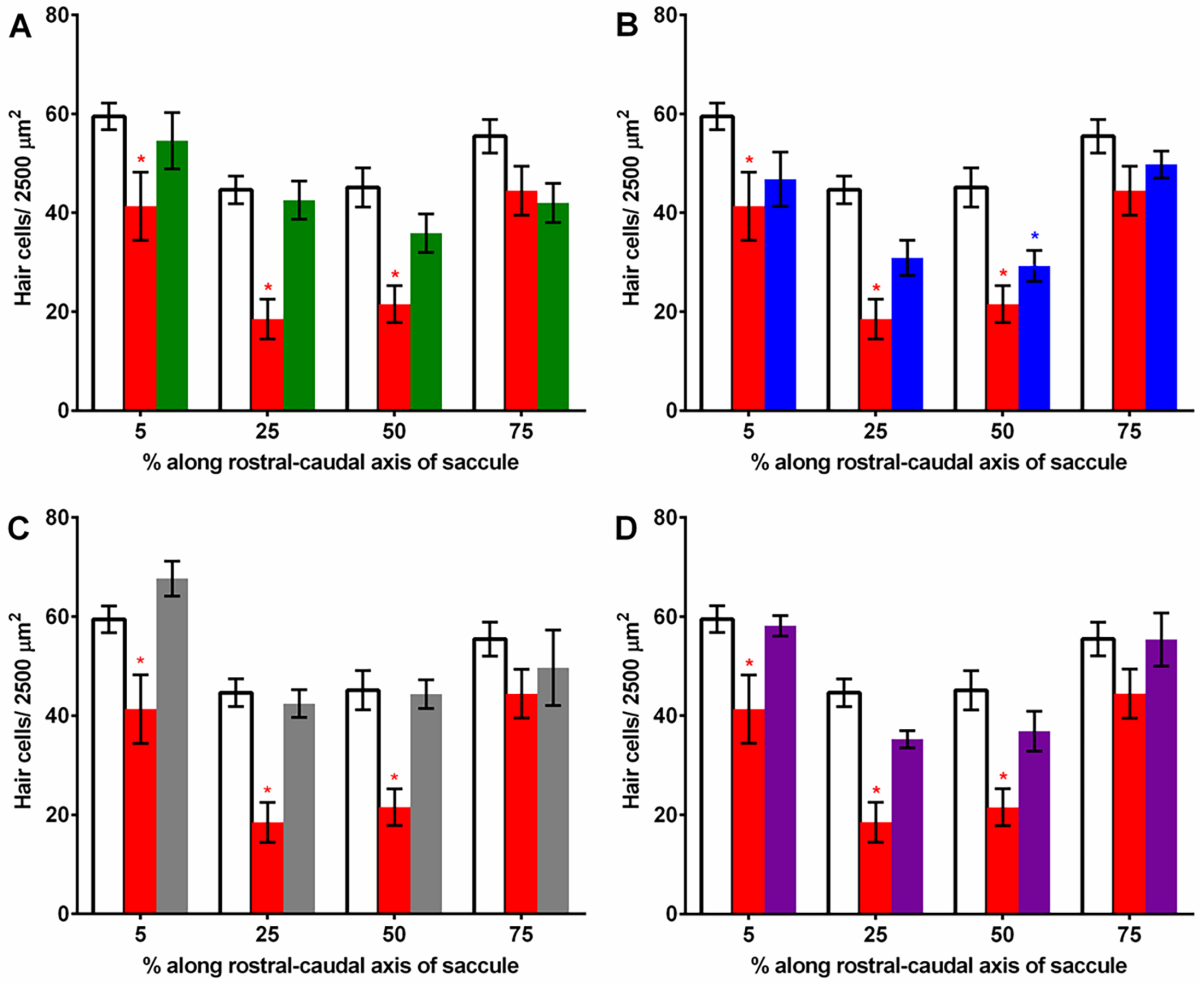
Mean (±SE) hair cell densities in four regions of the saccule treated with either a monofunctional platinum(II) complex, cisplatin or vehicle after 48 hours. A.-D. “5”, “25”, “50”, “75” designate length along the rostral-caudal axis. Cisplatin (red) treatment caused hair cell density reduction in the 5, 25 and 50% regions compared to vehicle (white) treatment. A. Pyriplatin (green) treatment caused no hair cell reduction. B. Phenanthriplatin (blue) treatment caused reduction only at 50%. C. [Pt(dien)Cl]^+^ (grey) and (D) [Pt(Et_2_dien)Cl]^+^ (purple) treatment caused no significant reduction in hair cell bundles. N = 8–15.

**Fig 8:**
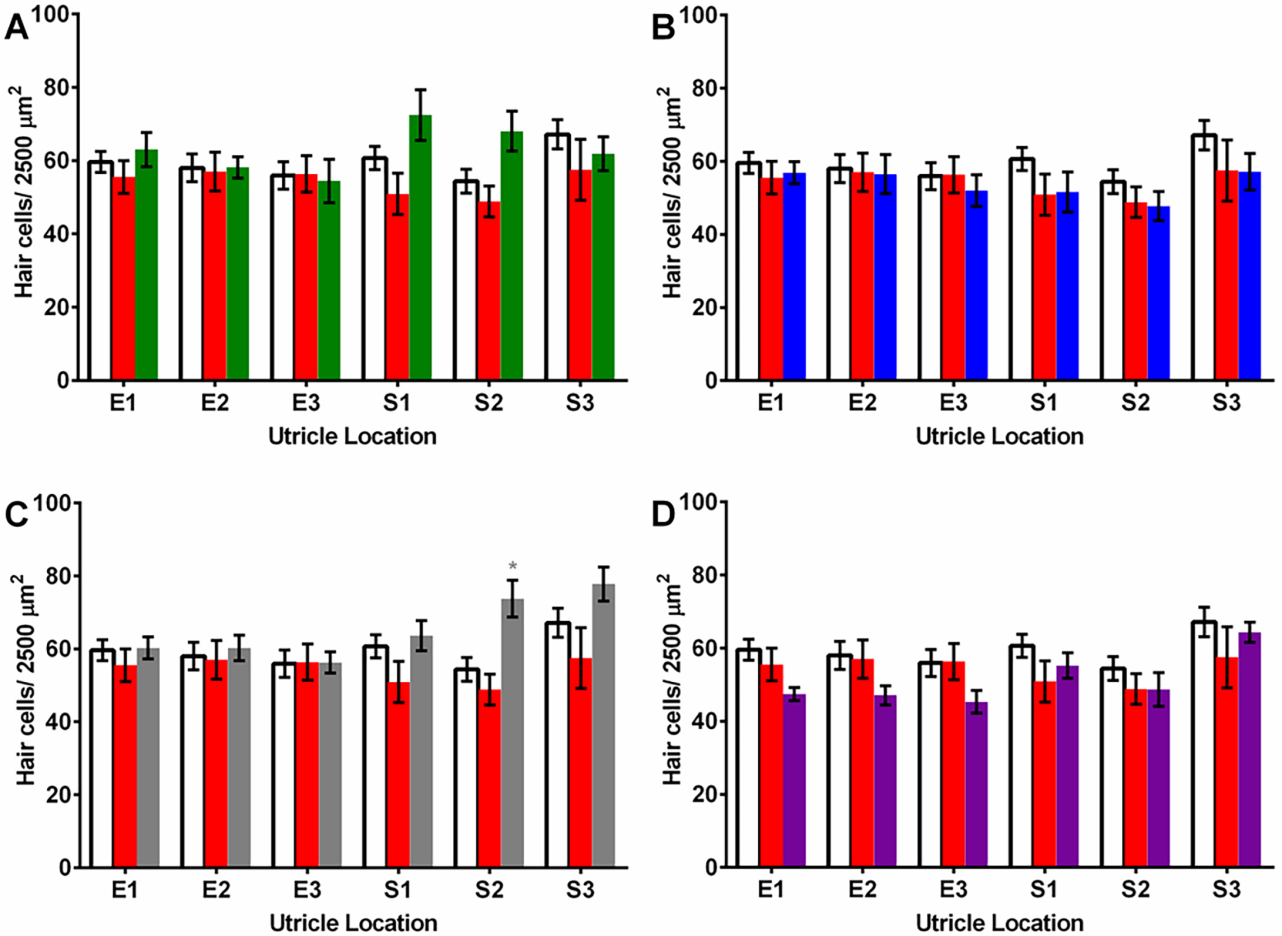
Mean (±SE) hair cell densities in six regions of the utricle treated with either a monofunctional platinum(II) complex, cisplatin or vehicle after 48 hours. A.-D. Cisplatin (red) treatment; vehicle (white) treatment; E1-E3: extrastriolar regions; S1-S3: striolar regions. A. Pyriplatin (green) treatment. B. Phenanthriplatin (blue) treatment. C. [Pt(dien)Cl]^+^ (grey) treatment had one elevated mean hair cell density count at S2. D. [Pt(Et_2_dien)Cl]^+^ (purple) treatment. N = 8–15.

We performed hair cell counts in the saccule and utricle to determine if platinum compound treatment caused damage to these auditory sensory structures. Our analysis of the saccule, the primary hearing end organ of the zebrafish [36–37], showed that at 24 hour post-injection, the compounds did not cause a significant effect on hair cell density. However, after 48 hours, cisplatin did cause a reduction in hair cell density in three regions, the 5% (69.5% of control), 25% (41.4% of control) and 50% (47.7% of control) locations along the rostral-caudal axis, and phenanthriplatin reduced hair cell counts in the 50% region (64.8% of control) (*p* < 0.05; Fig 7). In the utricle, an end organ that primarily functions in balance, hair cell density was not reduced compared to vehicle 24 hours following injection. Similarly, tissue samples taken from utricles 48 hours post-injection did not have reduced hair cell counts for any compound (Fig 8). These results suggest that cisplatin, and to a limited extent phenanthriplatin, can destroy hair cells in specific regions of the saccule, but that none of the platinum(II) complexes reduce hair cell counts in the utricle.

## Discussion

Here, we investigated whether platinum compounds with *cis* additive steric properties could have similar anti-cancer efficacy as cisplatin without causing the serious side-effects of hearing loss or auditory tissue damage associated with this FDA-approved chemotherapy drug. The heterocyclic-ligated monofunctional platinum(II) complexes, phenanthriplatin and pyriplatin, incorporate increased steric hindrance *cis* to the chloride leaving ligand attached to the platinum coordination site and have superior nuclear compartmental targeting, DNA guanine residue binding and anti-cancer efficacy [19, 22]. Pyriplatin and phenanthriplatin do not form DNA crosslinks and bend DNA like cisplatin, and this could mean that they signal through different downstream pathways and might produce reduced side-effects compared to cisplatin [19, 38–39]. This suggested to us that monofunctional triamine-ligated platinum(II) complexes with similar steric properties to heterocyclic-ligated compounds might reduce cancer cell-viability and have reduced auditory side-effects. We used the MTT, 5’-GMP binding and a cellular compartmental uptake assay along with a zebrafish inner ear model to compare pyriplatin and phenanthriplatin with [Pt(dien)Cl]^+^ and [Pt(Et_2_dien)Cl]^+^ and to assess how the addition of *cis* steric bulk affects their performance against cancer and their hearing and auditory tissue side-effects.

Increasing steric bulk *cis* to the chloride leaving ligand of a triamine-ligated platinum complex could improve nuclear targeting by reducing reactivity with sulfur containing ligands. The heterocyclic-ligated compound, pyriplatin, has an aromatic ring added *cis* to the chloride leaving ligand, and has similar reaction kinetics to either N-AcMet or 5’-GMP [19]. However, the addition of the larger heterocyclic phenanthridine ligand on phenanthriplatin shifts the reaction kinetics to favor 5’-GMP over N-AcMet. The triamine-ligated compound, [Pt(dien)Cl]^+^, which has little steric hindrance *cis* to both sides of the chloride leaving ligand reacts with thioethers faster than with 5’-GMP [23–24]. The addition of greater steric hindrance *cis* to one of the amine donor atoms in [Pt(Et_2_dien)Cl]^+^ still produces a reactivity towards N-AcMet faster than with 5’-GMP, but higher pH, as might be found in cellular systems, tends to reduce reactivity [25]. Notwithstanding theoretical predictions, our compartmental distribution data show that all test compounds exhibited similar uptake characteristics in both the cytosolic and nuclear compartments in every cell line (Fig 3, Fig 4). Thus, mechanisms other than kinetic reactivity to sulfur-containing ligands may play an important role in platinum uptake and nuclear compartmental targeting.

The steric features of triamine-ligated complexes could confer properties that increase their cellular uptake. It has been proposed that the phenanthridine ligand increases the hydrophobicity of phenanthriplatin relative to cisplatin and pyriplatin and is responsible for phenanthriplatin’s improved cellular uptake compared to these other compounds [19]. Both the copper transporters, CTR1 and CTR2, and the organic cation transporters, OCT1 and OCT2, have been implicated in the uptake of platinum(II) compounds [40–41]. Pyriplatin is efficiently taken up by both OCT1 and OCT2 [21–22]; however, [Pt(dien)Cl]^+^ and [Pt(Et_2_dien)Cl]^+^ transport-mediated uptake is currently not understood. Nonetheless, our cellular uptake data does not support the hypothesis that greater hydrophobicity from the inclusion of bridging substituents in [Pt(dien)Cl]^+^ and [Pt(Et_2_dien)Cl]^+^, or the addition of steric bulk *cis* to the chloride leaving ligand as in [Pt(Et_2_dien)Cl]^+^, pyriplatin or phenanthriplatin, increases cellular uptake efficiency (Fig 3). Instead, it is possible that transporter expression and activity is more important in driving cellular platinum uptake than the hydrophobic characteristics of these compounds.

Platinum(II) complexes with steric hindrance characteristics that promote 5’-GMP binding would be expected to more efficiently target DNA and have greater anti-cancer effect. Interestingly, our 5’-GMP rate kinetics data do not support the interpretation that increased guanosine residue binding affinity is correlated with decreased cellular viability. [Pt(dien)Cl]^+^, which has the smallest amount of steric hindrance around the chloride leaving ligand DNA binding site, had the least effect on cellular viability (Table 2). [Pt(Et_2_dien)Cl]^+^, which has considerably more steric hindrance proximate to the chloride ligand than [Pt(dien)Cl]^+^, has a much lower relative 5’-GMP binding rate, and yet in most cell lines reduces cellular viability much more effectively than [Pt(dien)Cl]^+^. Similarly, phenanthriplatin, which has significantly more steric hindrance proximate to the chloride leaving ligand than pyriplatin, has an almost identical 5’-GMP binding rate, but much stronger effect against cellular viability. Further, [Pt(Et_2_dien)Cl]^+^, which has a much slower relative rate constant than either phenanthriplatin and pyriplatin, reduced cellular viability to a value intermediate between the heterocyclic-ligated compounds in most cell lines tested (Fig 2; Table 2). Thus, we found that the relative rate of 5’-GMP binding is not an effective predictor of effect on cellular viability.

5'-GMP reactivity could also be affected by hydrogen bonding with the nonleaving ligands or by electronic effects. In some platinum(II) complexes, the presence of steric bulk in the form of methyl groups proximate to the leaving ligand can prevent the formation of hydrogen bonds with incoming 5’-GMP and slow reactivity considerably [42]. However, in the complexes investigated here, at least one NH_3_ or NH_2_ group is *cis* to the incoming position and, therefore, hydrogen bonding with 5'-GMP should still be possible. Electronic effects also can have an effect on reactivity: for example, introducing π conjugation greatly increases the reaction of monofunctional platinum complexes with thiourea [43]. Thus, the faster reactivity of phenanthriplatin relative to [Pt(Et_2_dien)Cl]^+^ could also be partly explained by an electronic effect as the addition of ethyl groups to [Pt(Et_2_dien)Cl]^+^ might diminish the positive charge on its platinum atom and considerably decrease 5’-GMP reactivity.

Addition of greater steric bulk *cis* to the chloride leaving ligand might promote transcription blockage and reduce cancer cell viability. Studies conducted with pyriplatin and phenanthriplatin using DNA templates show that these compounds prevent transcription by blocking RNA polymerase II [7,19–21]. The larger steric bulk of the phenanthridine ligand in phenanthriplatin should more effectively block the advance of RNA polymerase II and transcription than the smaller single heterocyclic ring substituent attached to pyriplatin. This interpretation is supported by our results as phenanthriplatin reduces cellular viability by a factor of at least 10 more than pyriplatin in our cell lines (Table 2). Early experimental work with [Pt(dien)Cl]^+^ found that unlike pyriplatin, the triamine compound had a negligible effect on DNA synthesis [44] and very limited effect against RNA transcription [21]. Our cellular viability results suggest that the ethylene bridges present between the amines in [Pt(dien)Cl]^+^ do not create sufficient steric hindrance to effectively block transcription (Table 2). However, the addition of two ethyl substituents to one of the amines in [Pt(dien)Cl]^+^ produces [Pt(Et_2_dien)Cl]^+^, which has a steric bulk profile similar to pyriplatin. We found that [Pt(Et_2_dien)Cl]^+^ reduces cellular viability in most cell lines more than pyriplatin suggesting that the introduction of steric bulk on one side of the triamine [Pt(dien)Cl]^+^ core molecule is responsible for the effect on viability possibly by increasing transcription blockage.

Monofunctional platinum(II) complexes could cause DNA damage and increase hair cell loss and hearing thresholds like cisplatin [2, 45]. Steric characteristics that promote guanine nucleotide binding could lead to more DNA damage and increase hair cell loss and hearing thresholds. However, our 5’-GMP binding rate data, which is in the order [Pt(dien)Cl]^+^ > pyriplatin > phenanthriplatin > [Pt(Et_2_dien)Cl]^+^ (Table 1), suggests that binding rate is not correlated with decreased hair cell density. Phenanthriplatin, which has an intermediate reaction rate, is the only monofunctional complex that reduced hair cell density in the saccule (50% region only) (Fig 7).

Similarly, we found that 5’-GMP reactivity rates are not correlated to threshold shifts. Phenanthriplatin produced the strongest mean (± S.E.) threshold shifts (13.3 ± 2.9 dB), followed closely by [Pt(dien)Cl]^+^ and [Pt(Et_2_dien)Cl]^+^ (12.0 ± 3.0 and 11.1 ± 2.5 dB, respectively), leaving pyriplatin (6.2 ± 2.1 dB) with the weakest effect (Fig 6). Cisplatin treatment not only produced significant threshold shifts (7.5 ± 2.5 dB) but also considerably more reduction in hair cell density (5, 25 and 50% regions of the saccule) (Fig 7). Therefore, the guanosine binding reactivity of the monofunctional complexes does not seem to be directly related to threshold shifts or hair cell loss.

It is possible that the steric properties of monofunctional complexes could affect transcription and increase threshold shifts without changing hair cell density. Monofunctional complexes with greater steric bulk can inhibit transcription (19, 21). The steric profile series, where the ligand attached *cis* to the chloride leaving ligand ranges from largest (phenanthridine) to no ligand attachment to an amine terminus is, for the monofunctional complexes: phenanthriplatin > pyriplatin > [Pt(Et_2_dien)Cl]^+^ > [Pt(dien)Cl]^+^ (Fig 1). However, as with the 5’-GMP binding rate data, our steric profile results are not correlated with hair cell density or mean threshold shifts. For example, pyriplatin, which has the second largest steric bulk profile, has the least effect on hearing thresholds, and did not reduce hair cell densities (Figs 5–8). These results suggest that genes undergoing transcription in hair cells could be disrupted to a similar degree by the four monofunctional compounds. As cisplatin causes DNA to bend and recruit apoptotic signaling proteins [11–16], initiation of hair cell death would be expected to produce threshold shifts; whereas, the monofunctional compounds may disrupt auditory gene transcription, and effect hearing, without causing hair cell apoptosis and reducing hair cell densities (Figs 5–8). Therefore, monofunctional complexes may affect DNA differently than cisplatin and cause threshold shifts without associated hair cell loss.

Additionally, monofunctional platinum(II) complexes could also bind proteins and change their function leading to hearing threshold shifts without changing hair cell density. Introduction of D-methionine and N-acetyl-cysteine in rodent and zebrafish lateral line models show that the presence of sulfur-ligand residues can prevent cisplatin-mediated hair cell death and threshold shifts [46–50]. The sulfur compounds, diethyldithiocarbamate and thiourea, have been used to reverse cisplatin and [Pt(dien)Cl]^+^ binding to glutathione and S-methylglutathione residues found in proteins [51]. Further, introduction of diethyldithiocarbamate prevented cisplatin nephrotoxicity, probably by removal of the platinum compound from protein bound sulfhydryl groups [52]. [Pt(dien)Cl]^+^, [Pt(Et_2_dien)Cl]^+^ and pyriplatin react more strongly with methionine residues than phenanthriplatin [42, 53–55], which could allow them to modulate proteins which are involved in auditory physiology. However, as phenanthriplatin has the greatest *cis* steric hindrance of the four monofunctional compounds, this could mean that phenanthriplatin protein binding, although potentially less frequent than the other three compounds, could cause greater functional effects on proteins than the other complexes. Thus, monofunctional compounds with specific steric bulk characteristics could bind to protein sulfur ligands and cause auditory physiological effects without permanently damaging hair cells.

Platinum compound uptake in zebrafish inner ear hair cells could occur through mechanotransduction channels as well as the membrane based transporters utilized by cancer cells. *In situ* hybridization studies have shown that there is expression of CTR1 but no detectable expression of OCT2 in zebrafish hair cells [56]. However, transporter inhibition in the zebrafish lateral line indicated that mechanotransduction is required for cisplatin uptake [57]. Mammalian hair cells express both OCT2 and CTR1 and separate blockage of these transporters prevented cisplatin uptake [58–60]. Zebrafish inner ear platinum uptake has not been characterized at this time, but the threshold shifts observed in our auditory study (Fig 5) suggests that the steric bulk characteristics of the monofunctional compounds do not bar their uptake. A rhodamine-cisplatin complex of considerably greater molecular weight than our platinum(II) complexes was able to enter lateral line hair cells via a mechanotransduction mechanism [57], which suggests that this mode of entry, if applicable in zebrafish inner ear hair cells, should readily allow their uptake. As we found no reduction of hair cell density in the utricle (Fig 8), this could mean that platinum uptake in this end organ is mechanistically different than in the saccule.

## Conclusion

Monofunctional platinum(II) complexes have properties unlike those found in the bifunctional anti-cancer drug cisplatin and could be able to target cancer cells without producing the ototoxic side-effects commonly associated with this chemotherapy compound. Addition of steric bulk *cis* to the coordinating center of a monofunctional DNA compound is one strategy that has emerged in the development of new chemotherapy drug candidates. Our results suggest that triamine-ligated complexes with this form of steric bulk can exhibit nuclear compartmental targeting and anti-cancer cell effects competitive with their heterocyclic-ligated counterparts. Further, although the triamine- and heterocyclic-ligated complexes produce auditory threshold shifts similar to cisplatin, unlike cisplatin, the monofunctional compounds produce virtually no effect on auditory hair cell density. This suggests that these complexes could act in a mechanistically different fashion than cisplatin, possibly through modulation of transcription or protein targets. Evidently, more research is needed to elucidate the mechanistic action of these monofunctional platinum(II) complexes in cancer and hearing.

## Abbreviations

dien: diethylenetriamine
Et_2_dien: N,N-diethyldiethylenetriamine
N-AcMet: N-acetylmethionine
5′-GMP: guanosine 5′-monophosphate
AEP: auditory evoked potentials

## References

[1] Langer T, am Zehnhoff-Dinnesen A, Radtke S, Meitert J, Zolk O. Understanding platinum-induced ototoxicity. Trends Pharmacol Sci. 2013;34: 458–69. doi: 10.1016/j.tips.2013.05.006 23769626 10.1016/j.tips.2013.05.006

[2] Karasawa T, Steyger PS. An integrated view of cisplatin-induced nephrotoxicity and ototoxicity. Toxicol Lett. 2015;237: 219–27. doi: 10.1016/j.toxlet.2015.06.012 26101797 10.1016/j.toxlet.2015.06.012 PMC4516600

[3] Landier W. Ototoxicity and Cancer Therapy. Cancer. 2016;122: 1647–58. doi: 10.1002/cncr.29779 26859792 10.1002/cncr.29779

[4] Lanvers-Kaminsky C, Zehnhoff-Dinnesen AA, Parfitt R, Ciarimboli G. Drug-induced ototoxicity: Mechanisms, Pharmacogenetics, and protective strategies. Clin Pharmacol Ther. 2017;101: 491–500. doi: 10.1002/cpt.603 28002638 10.1002/cpt.603

[5] Lovejoy KS, Lippard SJ. Non-traditional platinum compounds for improved accumulation, oral bioavailability, and tumor targeting. Dalton Trans. 2009;48: 10651–9. 10.1039/b913896j PMC2800312 20023892

[6] Jamieson ER, Lippard SJ. Structure, recognition, and processing of cisplatin-DNA adducts. Chem.Rev. 1999;99: 2467–2498. 11749487 10.1021/cr980421n

[7] Wang S, Zhang H, Malfatti M, de Vere White R, Lara PN Jr, Turteltaub K, Henderson P, Pan CX. Gemcitabine causes minimal modulation of carboplatin-DNA monoadduct formation and repair in bladder cancer cells. Chem Res Toxicol. 2010;23: 1653–1655. doi: 10.1021/tx1003547 21028869 10.1021/tx1003547 PMC2987236

[8] Alian OM, Azmi AS, Mohammad RM. Network insights on oxaliplatin anti-cancer mechanisms. Clinical and Translational Medicine 2012; 1: 1–7. 23369220 10.1186/2001-1326-1-26 PMC3560997

[9] Knox RJ, Friedlos F, Lydall DA, Roberts JJ. Mechanism of cytotoxicity of anticancer platinum drugs: evidence that cis-Diamminedichloroplatinum(II) and cis-Diammine (1,1-cyclobutanedicarboxylato) platinum(DII) differ only in the kinetics of their interaction with DNA. Cancer Res. 1986;46: 1972–9. 3512077

[10] Raymond E, Faivre S, Chaney S, Woynarowski J, Cvitkovic E. Cellular and molecular pharmacology of oxaliplatin. Mol Cancer Ther. 2002;1: 227–235. 12467217

[11] Cepeda V, Fuertes MA, Castilla J, Alonso C, Quevedo C, Pérez JM. Biochemical Mechanisms of Cisplatin Cytotoxicity. Anticancer Agents Med Chem. 2007;7: 3–18. 17266502 10.2174/187152007779314044

[12] Mandic A, Hansson J, Linder S, Shoshan MC. Cisplatin induces endoplasmic reticulum stress and nucleus-independent apoptotic signaling. J Biol Chem. 2003;278: 9100–6. doi: 10.1074/jbc.M210284200 12509415 10.1074/jbc.M210284200

[13] Nicotera TM, Hu BH, Henderson D. The caspase pathway in noise-induced apoptosis of the chinchilla cochlea. J Assoc Res Otolaryngol. 2003;4: 466–77. doi: 10.1007/s10162-002-3038-2 14534835 10.1007/s10162-002-3038-2 PMC3202741

[14] Jiang H, Sha SH, Forge A, Schacht J. Caspase-independent pathways of hair cell death induced by kanamycin in vivo. Cell Death Differ. 2006;13: 20–30. doi: 10.1038/sj.cdd.4401706 16021180 10.1038/sj.cdd.4401706 PMC1525047

[15] Yang X, Fraser M, Abedini MR, Bai T, Tsang BK. Regulation of apoptosis-inducing factor-mediated, cisplatin-induced apoptosis by Akt. Br J Cancer. 2008;98: 803–8. doi: 10.1038/sj.bjc.6604223 18283299 10.1038/sj.bjc.6604223 PMC2259169

[16] Feng H, Yin SH, Tang AZ. Blocking caspase-3-dependent pathway preserves hair cells from salicylate-induced apoptosis in the guinea pig cochlea. Mol Cell Biochem. 2011;353: 291–303. doi: 10.1007/s11010-011-0798-1 21503676 10.1007/s11010-011-0798-1

[17] Hollis LS, Sundquist WI, Burstyn JN, Heiger-Bernays WJ, Bellon SF, Ahmed KJ, Amundsen AR, Stern EW, Lippard SJ. Mechanistic studies of a novel class of trisubstituted platinum(II) antitumor agents. Cancer Res. 1991;51: 1866–75. 2004370

[18] Mariggio MA, Cafaggi S, Ottone M, Parodi B, Vanozzi MO, Mandys V, Viale M. Inhibition of cell growth, induction of apoptosis, and mechanism of action of the novel platinum compound cis-diaminechloro-[2-(diethylamino) ethyl 4-amino-benzoate, N^4^]–chloride platinum(II) monohydrochloride monohydrate. Invest New Drugs. 2004;22: 3–16. 14707490 10.1023/b:drug.0000006170.38419.c9

[19] Park GY, Wilson JJ, Song Y, Lippard SJ. Phenanthriplatin, a monofunctional DNA-binding platinum anticancer drug candidate with unusual potency and cellular activity profile. Proc Natl Acad Sci U S A. 2012;109: 11987–92. doi: 10.1073/pnas.1207670109 22773807 10.1073/pnas.1207670109 PMC3409760

[20] Zhu G, Myint MNZ, Ang WH. Monofunctional platinum-DNA adducts are strong inhibitors of transcription and substrates for nucleotide excision repair in live mammalian cells. Cancer Res. 2012;72: 790–800. doi: 10.1158/0008-5472.CAN-11-3151 22180496 10.1158/0008-5472.CAN-11-3151 PMC3271130

[21] Lovejoy KS, Todd RC, Zhang S, McCormick MS, D'Aquino JA, Reardon JT, Sancar A, Giacomini KM, Lippard SJ. cis-Diammine(pyridine)chloroplatinum(II), a mononfunctional platinum(II) antitumor agent: Uptake, structure, function, and prospects. Proc Natl Acad Sci U S A. 2008;105: 8902–7. doi: 10.1073/pnas.0803441105 18579768 10.1073/pnas.0803441105 PMC2449337

[22] Lovejoy KS, Serova M, Bieche I, Emami S, D'Incalci M, Broggini M, Erba E, Gespach C, Cvitkovic E, Faivre S, Raymond E, Lippard SJ. Spectrum of cellular responses to pyriplatin, a monofunctional cationic antineoplastic platinum(II) compound, in human cancer cells. Mol Cancer Ther. 2011;10: 1709–19. doi: 10.1158/1535-7163.MCT-11-0250 21750216 10.1158/1535-7163.MCT-11-0250 PMC3170455

[23] Djuran MI, Lempers ELM, Reedijk J. Reactivity of chloro-and aqua (diethylenetriamine) platinum (II) ions with glutathione, S-methylglutathione, and guanosine 5'-monophosphate in relation to the antitumor activity and toxicity of platinum complexes. Inorg. Chem. 1991;30: 2648–52.

[24] Barnham KJ, Djuran MI, Murdoch PD, Sadler PJ. Intermolecular displacement of S-bound L-methionine on platinum (II) by guanosine 5′-monophosphate: implications for the mechanism of action of anticancer drugs. J. Chem. Soc. Chem. Comm. 1994;6: 721–22.

[25] Williams KM, Gruner M, Gensheimer J, Wright A, Blair M, Autry SA, Hammer NI. Partial displacement of a triamine ligand from a platinum(II) complex after reaction with N-acetylmethionine. Inorg. Chim. Acta 2017;458: 163–70.

[26] Hoffman RE, Davies DB. Temperature Dependence of NMR Secondary References for D_2_O and (CD_3_)_2_SO Solutions. Magn. Reson. Chem. 1988;26: 523–25.

[27] Westerfield M. The Zebrafish Book: A Guide for the Laboratory Use of Zebrafish. 4^th^ ed. Eugene: Institute of Neuroscience, University of Oregon; 1994.

[28] Corwin JT, Bullock TH, Schweitzer J. The auditory brain stem response in five vertebrate classes. Electroencephalogr Clin Neurophysiol. 1982;54: 629–41. 6183096 10.1016/0013-4694(82)90117-1

[29] Kenyon TN, Ladich F, Yan HY. A comparative study of hearing ability in fishes: the auditory brainstem response approach. J Comp Physiol A. 1998;182: 307–18. 9528109 10.1007/s003590050181

[30] Smith ME, Kane AS, Popper AN. Noise-induced stress response and hearing loss in goldfish (*Carassius auratus*). J. Exp. Biol. 2004;207: 427–435. 14691090 10.1242/jeb.00755

[31] Smith ME, Kane AS, Popper AN. Acoustical stress and hearing sensitivity in fishes: does the linear threshold hypothesis hold water? J. Exp. Biol. 2004;207: 3591–3602. doi: 10.1242/jeb.01188 15339955 10.1242/jeb.01188

[32] Smith ME, Coffin AB, Miller DL, Popper AN. Anatomical and functional recovery of the goldfish (*Carassius auratus*) ear following noise exposure. J. Exp. Biol. 2006;209: 4193–4202. doi: 10.1242/jeb.02490 17050834 10.1242/jeb.02490

[33] Sun H, Lin CH, Smith ME. Growth hormone promotes hair cell regeneration in the zebrafish (*Danio rerio*) inner ear following acoustic trauma. PLoS ONE 6(11): e28372 doi: 10.1371/journal.pone.0028372 22140580 10.1371/journal.pone.0028372 PMC3227666

[34] Oxman DS, Barnett-Johnson R, Smith ME, Coffin AB, Miller DD, Josephson R, Popper AN. The effect of vaterite deposition on otolith morphology, sound reception and inner ear sensory epithelia in hatchery-reared chinook salmon (*Oncorhynchus tshawytscha*). Can. J. Fish. Aquatic Sci. 2007;64: 1469–1478.

[35] Schuck JB, Smith ME. Cell proliferation follows acoustically-induced hair cell bundle loss in the zebrafish saccule. Hear Res. 2009;253: 67–76. doi: 10.1016/j.heares.2009.03.008 19327392 10.1016/j.heares.2009.03.008 PMC2810637

[36] Popper AN, Fay RR. Sound detection and processing by fish: critical review and major research questions. Brain Behav Evol. 1993;41: 14–38. doi: 10.1159/000113821 8431753 10.1159/000113821

[37] Wang J, Song Q, Yu D, Yang G, Xia L, Su K, Shi H, Wang J, Yin S. Ontogenetic development of the auditory sensory organ in zebrafish (*Danio rerio*): changes in hearing sensitivity and related morphology. Sci Rep. 2015;5: 15943 doi: 10.1038/srep15943 26526229 10.1038/srep15943 PMC4630651

[38] Wang D, Zhu G, Huang X, Lippard SJ., X-ray structure and mechanism of RNA polymerase II stalled at an antineoplastic monofunctional platinum-DNA adduct., Proc Natl Acad Sci U S A. 2010;107: 9584–9. doi: 10.1073/pnas.1002565107 20448203 10.1073/pnas.1002565107 PMC2906855

[39] Gregory MT, Park GY, Johnstone TC, Lee YS, Yang W, Lippard SJ. Structural and mechanistic studies of polymerase η bypass of phenanthriplatin DNA damage. Proc Natl Acad Sci U S A. 2014;111: 9133–8. doi: 10.1073/pnas.1405739111 24927576 10.1073/pnas.1405739111 PMC4078841

[40] Johnstone TC, Park GY, Lippard SJ. Understanding and improving platinum anticancer drugs—phenanthriplatin. Anticancer Res. 2014;34: 471–76. 24403503 PMC3937549

[41] Johnstone TC, Suntharalingam K, Lippard SJ. Third row transition metals for the treatment of cancer. Phil.Trans.R.Soc.A 2015;373: 1–12. 10.1098/rsta.2014.0185 PMC4342973 25666060

[42] Sandlin RD, Whelan CJ, Bradley MS, Williams KM. Effects of amine ligand bulk and hydrogen bonding on the rate of reaction of platinum(II) diamine complexes with key nucleotide and amino acid residues. Inorg. Chim. Acta. 2012;391: 135–40. 10.1016/j.ica.2012.05.008 PMC3418705 22904573

[43] Jaganyl D, Hofmann A, van Eldik R. Controlling the lability of square-planar Pt(II) complexes through electronic communication between π-acceptor ligands. Angew. Chem. 2001;40: 1680–1683. 11353478

[44] Pinto AL, Lippard SJ. Sequence-dependent termination of in vitro DNA synthesis by cis- and trans-diamminedichloroplatinum (II). Proc. Natl. Acad. Sci. USA. 1985;82: 4616–19. 3895221 10.1073/pnas.82.14.4616 PMC390436

[45] Paken J, Govender CD, Pillay M, Sewram V. Cisplatin-Associated Ototoxicity: A Review for the Health Professional. J Toxicol. 2016;2016: 1809394 doi: 10.1155/2016/1809394 28115933 10.1155/2016/1809394 PMC5223030

[46] Giordano P, Lorito G, Ciorba A, Martini A, Hatzopoulos S. Protection against cisplatin ototoxicity in a Sprague-Dawley rat animal model. Acta Otorhinolaryngol Ital. 2006;26: 198–207. 18236636 PMC2639998

[47] Campbell KC, Meech RP, Klemens JJ, Gerberi MT, Dyrstad SS, Larsen DL, Mitchell DL, El-Azizi M, Verhulst SJ, Hughes LF. Prevention of noise- and drug-induced hearing loss with D-methionine. Hear Res. 2007;226: 92–103. doi: 10.1016/j.heares.2006.11.012 17224251 10.1016/j.heares.2006.11.012

[48] Lorito G, Hatzopoulos S, Laurell G, Campbell KC, Petruccelli J, Giordano P, Kochanek K, Sliwa L, Martini A, Skarzynski H. Dose-dependent protection on cisplatin-induced ototoxicity—an electrophysiological study on the effect of three antioxidants in the Sprague-Dawley rat animal model. Med Sci Monit. 2011;17: BR179–186. doi: 10.12659/MSM.881894 21804453 10.12659/MSM.881894 PMC3539615

[49] Clifford RE, Coleman JK, Balough BJ, Liu J, Kopke RD, Jackson RL. Low-dose D-methionine and N-acetyl-L-cysteine for protection from permanent noise-induced hearing loss in chinchillas. Otolaryngol Head Neck Surg. 2011;145: 999–1006. doi: 10.1177/0194599811414496 21750343 10.1177/0194599811414496

[50] Coffin AB, Williamson KL, Mamiya A, Raible DW, Rubel EW. Profiling drug-induced cell death pathways in the zebrafish lateral line. Apoptosis. 2013;18: 393–408. doi: 10.1007/s10495-013-0816-8 23413197 10.1007/s10495-013-0816-8 PMC3627356

[51] Lempers ELM, Reedijk J. Reversibility of binding of cisplatin-methionine in proteins by diethyldithiocarbamate or thiourea: a study with model adducts. Inorg. Chem., 1990;29: 217–222.

[52] Borch RF, Pleasants ME. Inhibition of cis-platinum nephrotoxicity by diethyldithiocarbamate rescue in a rat model. Proc Natl Acad Sci U S A. 1979;76: 6611–4. 230514 10.1073/pnas.76.12.6611 PMC411916

[53] Williams KM, Chapman DJ, Massey SR, Haare C. Interaction of N-acetylmethionine with a non-C_2_-symmetrical platinum diamine complex. J. Inorg. Biochem. 2005;99: 2119–26. doi: 10.1016/j.jinorgbio.2005.07.010 16129491 10.1016/j.jinorgbio.2005.07.010

[54] Sandlin RD, Starling MP, Williams KM. A bulky platinum triamine complex that reacts faster with guanosine 5′-monophosphate than with N-acetylmethionine. J. Inorg. Biochem. 2010;104: 214–6. doi: 10.1016/j.jinorgbio.2009.10.013 19906431 10.1016/j.jinorgbio.2009.10.013 PMC2815243

[55] Williams KM, Dudgeon RP, Chmely SC, Robey SR. Reaction of platinum(II) diamine and triamine complexes with selenomethionine. Inorganica chimica acta. 2011;368: 187–193. doi: 10.1016/j.ica.2011.01.002 21516209 10.1016/j.ica.2011.01.002 PMC3079887

[56] McDermott BM, Baucom JM, Hudspeth AJ. Analysis and functional evaluation of the hair-cell transcriptome. Proc Natl Acad Sci U.S.A. 2007;104: 11820–11825. doi: 10.1073/pnas.0704476104 17606911 10.1073/pnas.0704476104 PMC1905926

[57] Thomas AJ, Hailey DW, Stawicki TM, Wu P, Coffin AB, Rubel EW, Raible DW, Simon JA, Ou HC. Functional mechanotransduction is required for cisplatin-induced hair cell death in the zebrafish lateral line. J Neurosci. 2013;33: 4405–14. doi: 10.1523/JNEUROSCI.3940-12.2013 23467357 10.1523/JNEUROSCI.3940-12.2013 PMC3666553

[58] Ciarimboli G, Deuster D, Knief A, Sperling M, Holtkamp M, Edemir B, Pavenstadt H, Lanvers-Kaminsky C, am Zehnhoff-Dinnesen A, Schinkel AH, Koepsell H, Jurgens H, Schlatter E. Organic cation transporter 2 mediates cisplatin-induced oto- and nephrotoxicity and is a target for protective interventions. Am J Pathol. 2010;176: 1169–1180. doi: 10.2353/ajpath.2010.090610 20110413 10.2353/ajpath.2010.090610 PMC2832140

[59] More SS, Akil O, Ianculescu AG, Geier EG, Lustig LR, Giacomini KM. Role of the copper transporter, CTR1, in platinum-induced ototoxicity. J Neurosci. 2010;30: 9500–9509. doi: 10.1523/JNEUROSCI.1544-10.2010 20631178 10.1523/JNEUROSCI.1544-10.2010 PMC2949060

[60] Ding D, He J, Allman BL, Yu D, Jiang H, Seigel GM, Salvi RJ. Cisplatin ototoxicity in rat cochlear organotypic cultures. Hear Res. 2011;282: 196–203. doi: 10.1016/j.heares.2011.08.002 21854840 10.1016/j.heares.2011.08.002 PMC3230738

